# Supplementary material for: Nested PCR to optimize rpoB metabarcoding for low-concentration and host-associated bacterial DNA
Source: Microbiol Spectr. 2025 Aug 12;13(9):e01417-25. doi: 10.1128/spectrum.01417-25 (PMC12403564; doi:10.1128/spectrum.01417-25)
Supplement: Supplemental material — Fig. S1 to S8. [file spectrum.01417-25-s0001.pdf]

Supplemental Figure S1

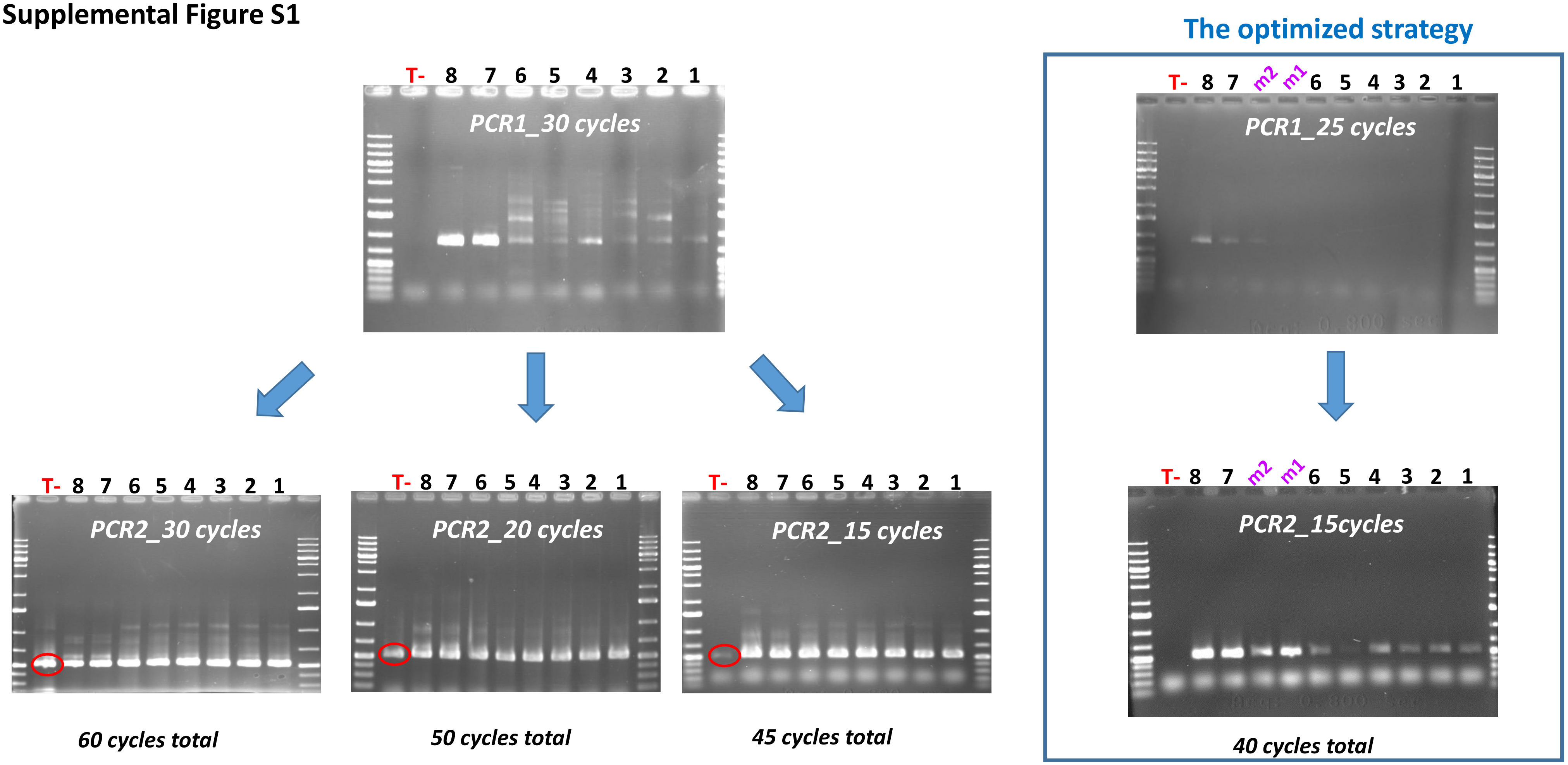

Supplemental Figure S1. Agarose gel electrophoresis of *rpoB* amplicons obtained using various nested PCR protocols with different numbers of PCR1 and PCR2 cycles. For each condition, the number of cycles used in PCR1 and PCR2 is indicated. The total cycle number (PCR1 + PCR2) is shown below the gel picture. A red circle highlights an amplification band observed in the negative control. Sample identities by lane are as follows: lanes 1, 2, 3, 4, 5, 6 are insect DNA samples; lanes 7, 8 are bacterial DNA samples; lanes m1 and m2 are mock_8sp and mock_8sp_log samples, respectively (mocks are only amplified using a 40-cycle protocol); lanes T- (negative control) is sterile water.

Supplemental Figure S2

Number of genomes:	47069
Amplified sequences:	41454
% of amplified sequences:	88.07%

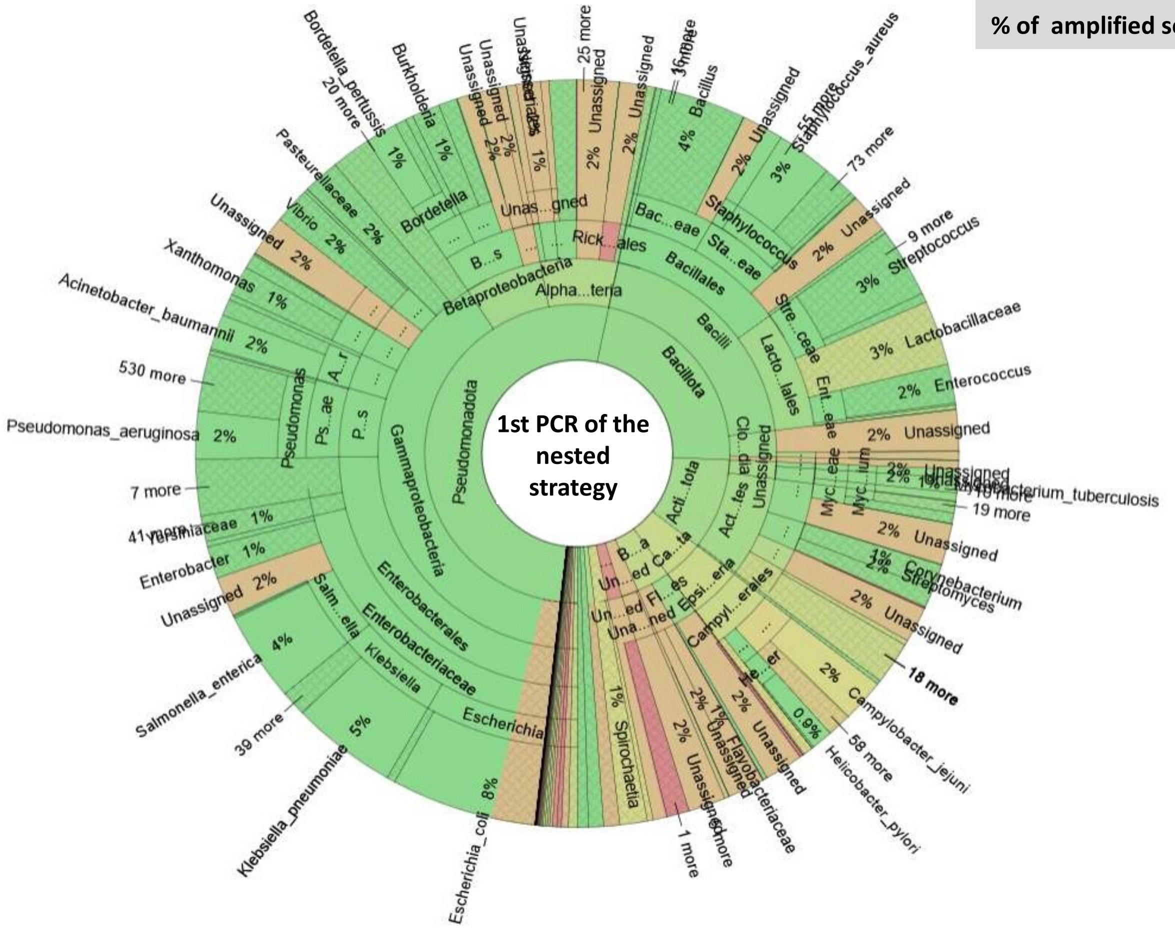

Supplemental Figure S2. Krona representations of the bacterial taxa potentially amplified by the outer primers used in the first round of PCR in the nested *rpoB* PCR strategy. The *in silico* EcoPCR v1.0.1 tool was used on the 47,069 *rpoB* sequences stored in the *rpoB* database, with two mismatches allowed. The Krona graph displays the major taxa potentially amplified by the outer *rpoB* primers (in green). Taxa that are not amplified are shown in pink. For more details on the taxa covered by the two PCR strategies, all Krona visualizations are accessible via the interactive link: https://github.com/geraldinepascal/RPOB_paper/tree/main/krona_figure.

Supplemental Figure S3

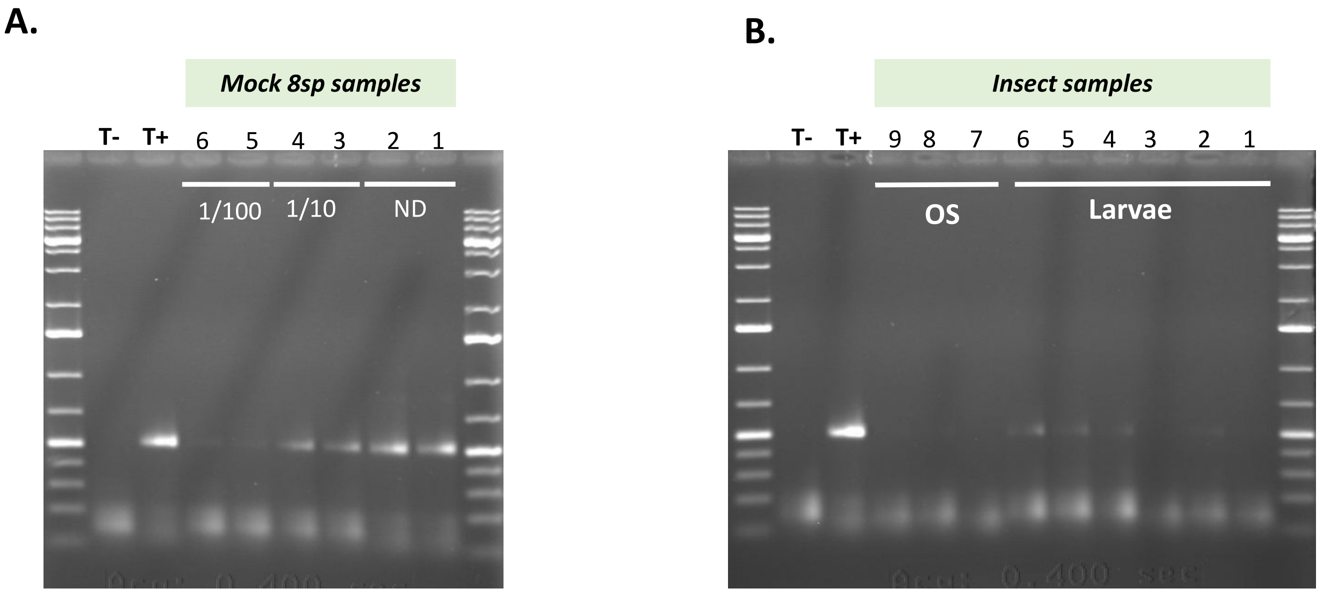

Supplemental Figure S3. Agarose gel electrophoresis of *rpoB* amplicons from mock_8sp (**panel A**) and insect samples (**panel B**) amplified by single-step PCR (40 cycles). **A.** The dilutions of the mock samples are indicated on the agarose gel, below the wells. For each dilution, two technical replicates are shown. ND: not diluted. T+ (positive control) : DNA from *Xenorhabdus*; T- (negative control): sterile water. **B.** The larval samples correspond to several biological replicates of the third developmental (lanes 1, 2, 3) and the fifth developmental stage (lanes 4, 5, 6) of the caterpillar. The OS (oral secretions) samples correspond to three independent PCR replicates (lanes 7, 8 and 9). T+ (positive control) : DNA from *Xenorhabdus*; T- (negative control): sterile water.

Supplemental Figure S4

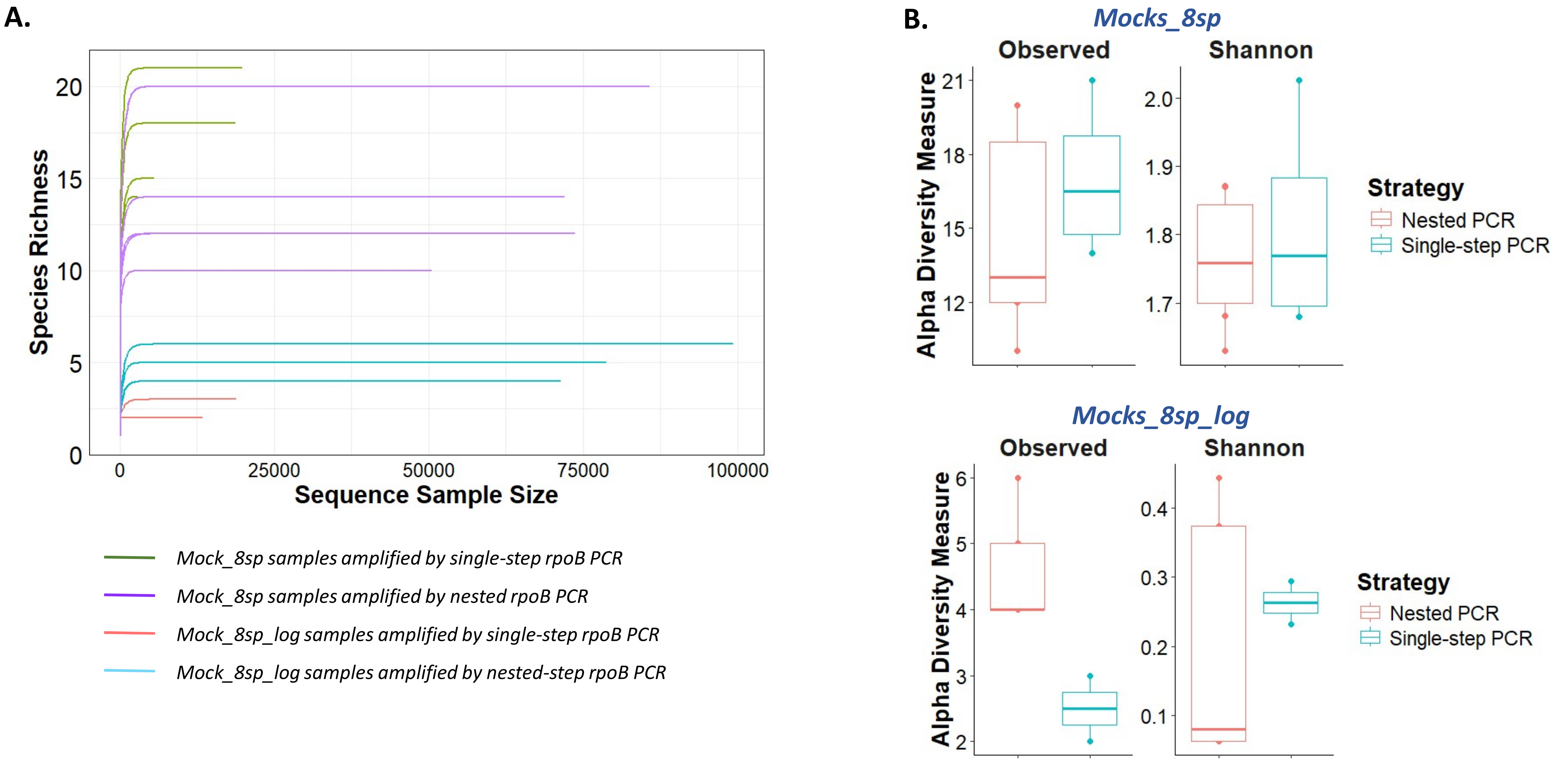

Supplemental Figure S4. Diversity analysis of commercial mock communities amplified with single-step and nested rpoB PCR strategies. A. Rarefaction curves for the metabarcoding sequences of the different commercial mock samples amplified by the single-step or nested *rpoB* PCR strategy. For each sample, species richness is shown on the *y*-axis and the number of sequences is shown on the *x*-axis. The ggrare function was used to generate the rarefaction curves (https://rdrr.io/github/gauravsk/ranacapa/man/ggrare.html). **B.** Comparison of alpha diversity indices (Observed and Shannon) between amplification strategies using the "plot_richness" function from the MicrobiotaProcess package. Statistical comparisons between strategies were performed using pairwise Wilcoxon rank-sum tests (Benjamini-Hochberg correction) on richness estimates obtained via the estimate_richness function from the MicrobiotaProcess package. No significant differences in alpha diversity were detected between strategies.

Supplemental Figure S5

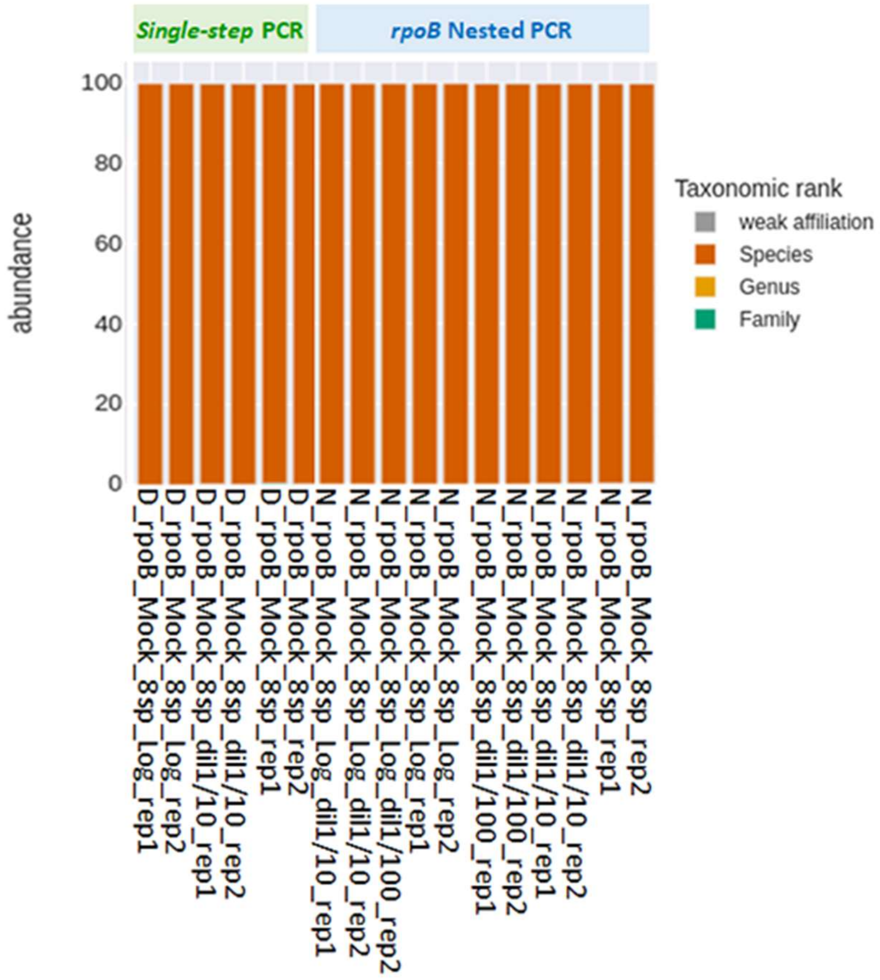

Supplemental Figure S5. Plot showing the relative abundance of ASVs at different taxonomic ranks for the various commercial mock samples following amplification by the single-step or nested *rpoB* PCR strategy. Two custom scripts were used to generate the plot: add_multiaffi_to_abd_table.py (https://github.com/geraldinepascal/RPOB_paper/blob/main/FROGS_analysis_results/scripts/add_multiaffi_to_abd_table.py) and plot_taxo_ranks.py (https://github.com/geraldinepascal/RPOB_paper/blob/main/FROGS_analysis_results/scripts/plot_taxo_ranks.py).

Supplemental Figure S6

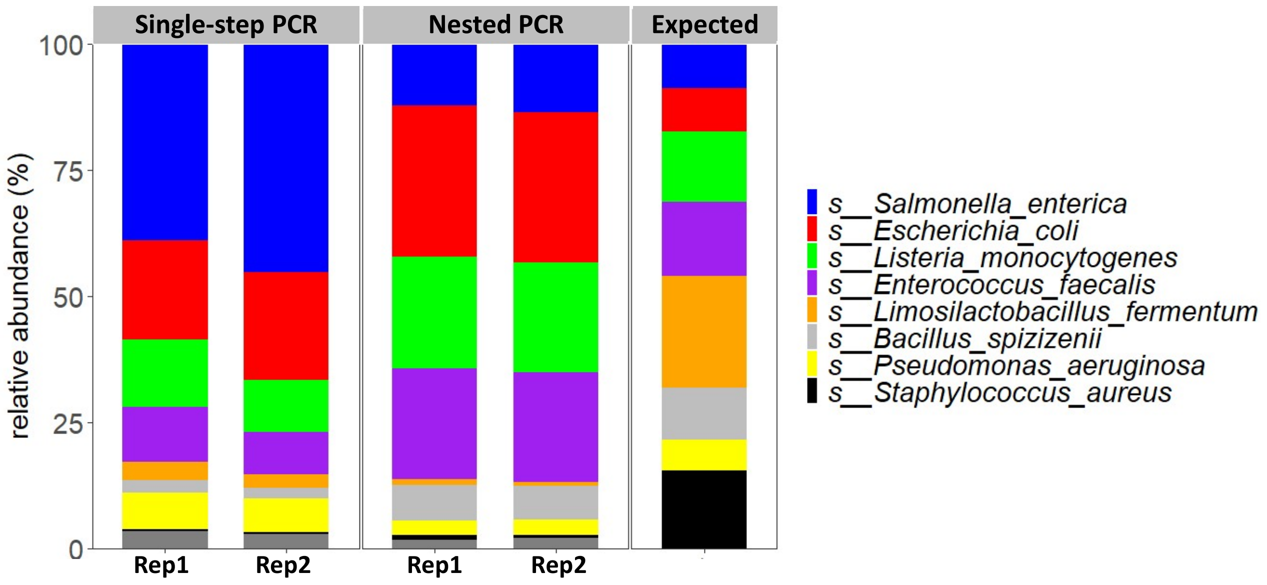

Supplemental Figure S6. Histogram plot of bacterial composition showing the relative abundances of ASVs at species level in the non-diluted commercial mock_8sp sample, amplified by the single-step or nested *rpoB* PCR strategy. Two technical replicates (rep1 and rep2) were performed, with the last bar indicating the expected composition.

Supplemental Figure S7

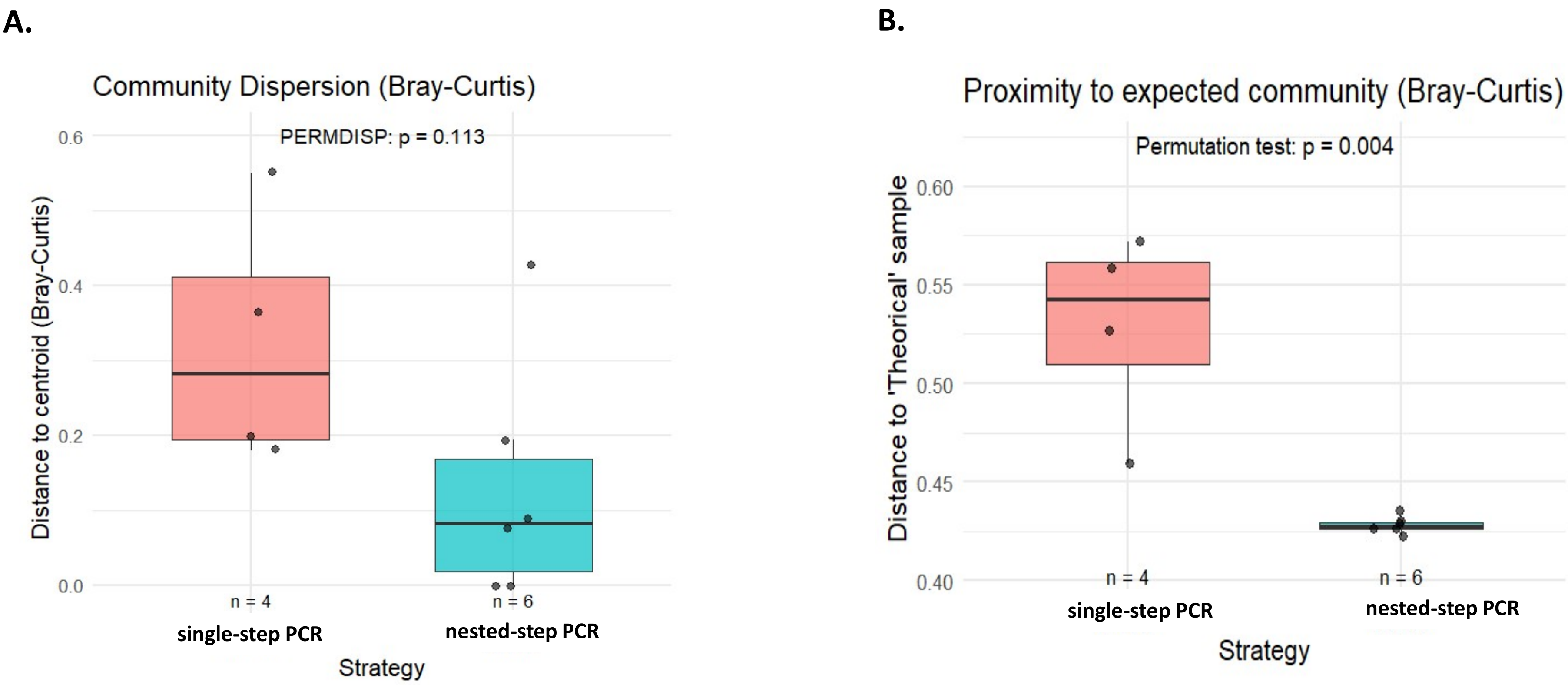

Supplemental Figure S7. Comparison of reproducibility and accuracy of microbial profiles between single-step and nested PCR strategies on mock microbial communities (mock_8sp not diluted, diluted at 1/10 or 1/100). A. Community dispersion measured by Bray-Curtis distances to group centroids. Each dot represents a replicate (n = 4 for single-step PCR, n = 6 for nested PCR). Although not statistically significant (PERMDISP: F = 3.25, p = 0.113), a lower dispersion is observed in the Nested strategy, suggesting higher consistency among replicates. **B.** Comparison of Bray-Curtis distances between experimental samples and the expected community profile across two PCR strategies: single-step PCR and nested PCR. Lower distances indicate greater similarity to the expected composition. Boxplots show the distribution of replicate distances per strategy (n = 4 for single-step PCR, n = 6 for nested PCR). The nested PCR strategy produced significantly closer profiles to the expected community (Permutation test, p = 0.004).

Supplemental Figure S8

A. OS samples

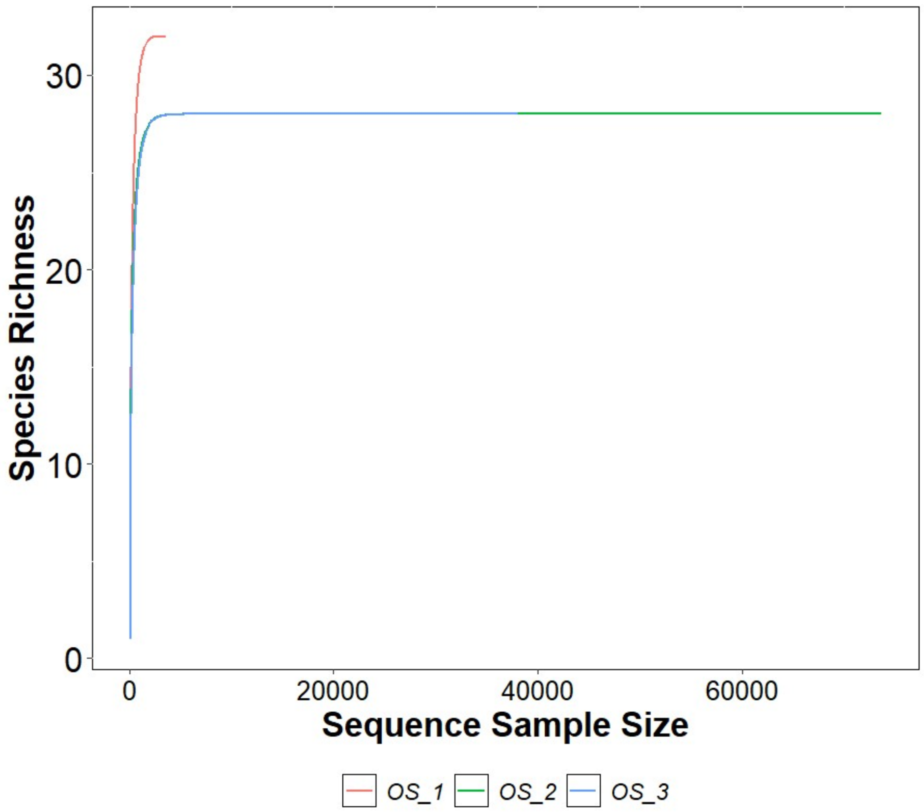

B. Larval samples

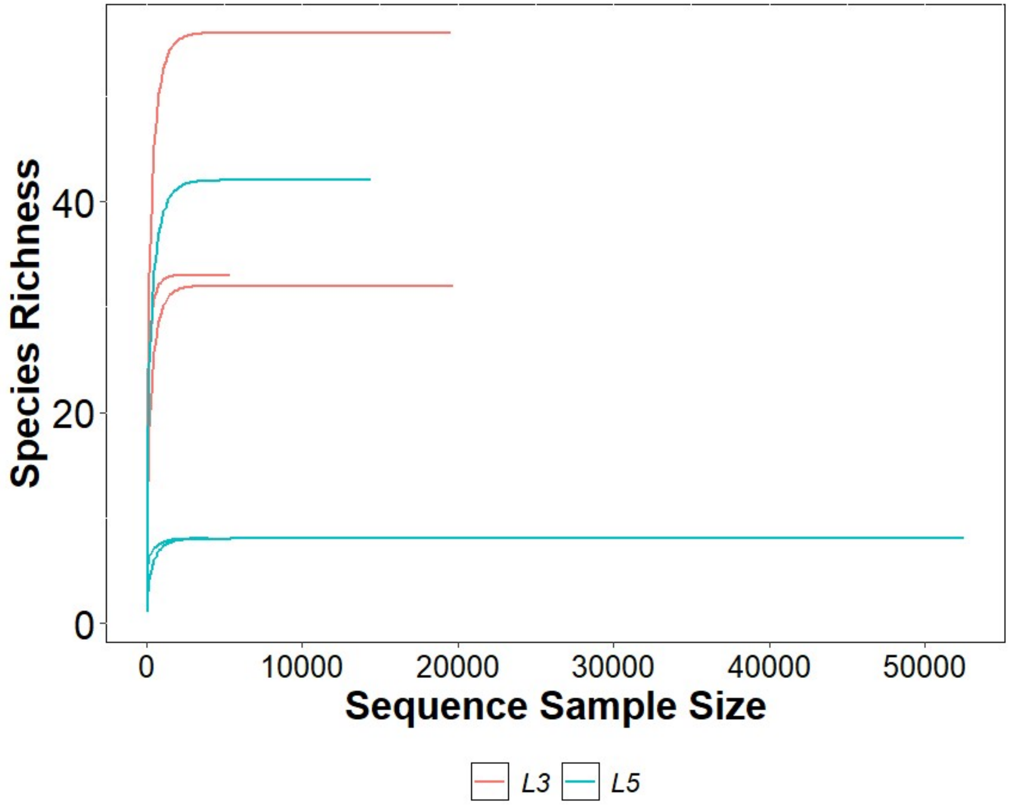

Supplemental Figure S8. Rarefaction curves for the metabarcoding sequences of *S. frugiperda* OS samples ***(panel A)*** and *S. frugiperda* demi-larva samples ***(panel B)*** subjected to amplification by the single-step or nested *rpoB* PCR strategy. The OS samples correspond to three independent PCR replicates. The larval samples correspond to several biological replicates: L3 is the third developmental stage, and L5 is the fifth developmental stage of the caterpillar. For each sample, species richness is displayed on the *y*-axis and the number of sequences on the *x*-axis. The ggrare function was used to generate the rarefaction curves (https://rdrr.io/github/gauravsk/ranacapa/man/ggrare.html).
