# Supplementary material for: Nested PCR to optimize rpoB metabarcoding for low-concentration and host-associated bacterial DNA
Source: Microbiol Spectr. 2025 Aug 12;13(9):e01417-25. doi: 10.1128/spectrum.01417-25 (PMC12403564; doi:10.1128/spectrum.01417-25)
Supplement: Table S2 — Parameters used for FROGS analysis. [file spectrum.01417-25-s0003.docx]

**Supplemental TableS2**: FROGS 4.1.0 parameters used for this study for *rpoB* amplicons from larvae, oral secretion and mock samples.

| **Tool** | ***rpoB*** |
| --- | --- |
| **preprocessing** | Illumina  --min-amplicon-size 300 --max-amplicon-size 590 –five-prim-primer GGYTWYGAAGTNCGHGACGTDCA --three-prim-primer TKATGGGYKCVAACATGCARCGTCA  --R1-size 300 --R2-size 300 |
| **clustering** | --fastidious --distance 1 |
| **remove_chimera** | default |
| **cluster_filters** | --min-abundance 0.00005 |
| **taxonomic_affiliation** | --rdp  --reference rpoB_bacteria_NCBI_refseq_genome_complete_and_chromosome_20240707.fasta |
